# Kind Words and Suggestions

**Published:** 1873-03

**Authors:** 


*Kind Words and Suggestions.*

"The plain truth is, that the profession has fallen very low in every sense of the word, and is destined to go still lower. Your journal is much needed in every section of this State, and the topics should be those which bear upon medical education—the intercourse to be observed by physicians towards each other; and the many dishonorable, mean and low practices now in vogue should be handled with the intensest severity. Under-charging—not directly, but by every kind of indirection known to the profession; evil-speaking, or the sly inuendo, and a thousand and one other acts that deserve the severest kind of castigation—these are subjects that should find a large space in all the medical periodicals of the day; and I hope you will take them in hand and give us a paper in each number upon some of these subjects during the present year."

We think our friend is right. The profession is being, in many places, reduced to a trade. Measures and schemes for personal advancement, at the sacrifice of truth, honor and ethics, are so common among medical men of to-day as to almost paralyze the efforts of those who seek to correct them. It would seem that, in spite of principle—in spite of the great interests involved—the profession is being hurried to hopeless ruin by a general apostacy of its members, alone bent upon private gain and personal aggrandizement.

Medical journals should warn the profession of the dangers ahead. We have tried to do this, and rejoice that so many have said—"You have done your duty." Our columns are open for discussion of questions touching the vital interests of the profession—of principles which must alone give exaltation and glory to the brotherhood.

*Tennessee.*—"Let nothing discourage you. The RECORD is regarded by all who have seen it in this section the best they have ever read. I will send you several subscribers soon."

We thank our friend for his kind words. Many letters of similar "tone" have been received. These friends have gone to work and have already sent us a number of subscribers. We trust all will do likewise.

*North Carolina.*—"Put the journal up to five dollars at once, for it is amply worth the money."

Many, like the correspondent above, have urged us to raise the price of subscription. We take pleasure in saying, however, that the present price will amply satisfy the requirements of its publication if our friends, one and all, will promptly remit us their subscriptions, and aid us by extending its circulation among their friends. Will not the friends of the RECORD—those who desire to see it improved month by month and year by year—go to work *actively* for it? In proportion as they succeed, so will be our ability to enlarge and improve it. Help the journal, and it will help you.

*Georgia.*—"The RECORD is a great improvement over the *Companion.* It is all the profession of the South could expect at the price and under all the circumstances. As a matter of pride, however, we would be glad to know what would induce you to increase its size sixteen pages, and put it on still better paper. We desire to see it the prettiest as well as the most useful and practical."

We need no inducement to cause us to bring the journal up to all the profession desire but an increase in the subscription list, which can be easily done if each subscriber will send in a new subscriber. If they will do this, the RECORD can be made just what its friends wish. The matter is in their hands. It can be accomplished by an hour's work by each of its present subscribers. Will they not try?

*Georgia.*—"I have not received the February number of the RECORD. Please send it at once—I can't do without it. I would not fail to get it for the subscription price."

While we take pleasure in sending the number requested, yet we must say our mailing department is so thoroughly perfected that no mistake can occur in it. Journals may not reach their destination from other causes, and we ask our friends to inform their postmasters that they expect the RECORD about certain times, and to hold them responsible. We sincerely thank our friend for his appreciation of the RECORD.

